# Progression of corneal thinning and melting after collagen cross-linking for keratoconus

**DOI:** 10.22336/rjo.2024.44

**Published:** 2024

**Authors:** Shreesha Kumar Kodavoor, Suvarna Chaji Sugali, Gopinathan Selvaraju, Ramamurthy Dandapani

**Affiliations:** 1 The Eye Foundation, Coimbatore, India; 2 The Eye Foundation, Tirupur, India

**Keywords:** collagen cross-linking, post-CXL corneal thinning, keratoconus, CXL = collagen cross-linking, IVF = in vitro fertilization, DALK = deep anterior lamellar keratoplasty, PKP = penetrating keratoplasty, KC = keratoconus, EMR = electronic record media, AS-OCT = anterior segment ocular coherence tomography, DM = diabetes mellitus, MMP = matrix metalloproteinases, BCVA = best corrected visual acuity, TCT = thinnest corneal thickness, CF = counting figures

## Abstract

**Objective:**

To study the aetiology, diverse clinical manifestations, therapeutic interventions, and prognoses in patients with corneal thinning after collagen cross-linking (CXL) treatment.

**Methods:**

a retrospective observational study of all patients presenting with corneal thinning after collagen cross-linking, in a tertiary eye care hospital in south India from 2011 to 2017. Preoperative details were noted. Patients who presented with corneal thinning were subjected to investigative measures to assess thinning, received appropriate management, and underwent follow-up evaluations.

**Results:**

Among the 12 patients, 8 were female and 4 were male, all of whom had undergone standard classical CXL. The duration between CXL and corneal melting/thinning onset varied from 5 to 12 years. Notably, among female patients, 2 were pregnant, 2 were lactating mothers, 1 was undergoing in vitro fertilization (IVF) treatment, and 1 had comorbid diabetes mellitus and hyperthyroidism. Clinical examination revealed corneal thinning accompanied by hypopyon in 1 patient, corneal perforation with shallow anterior chamber in 1 patient, and epithelial defect with crystalline deposit in another patient, the remaining patients exhibited corneal thinning. Corneal thinnest corneal thickness measurements ranged from 212 to 351 µm. Treatment approaches included penetrating keratoplasty (PKP) in 2 patients, deep anterior lamellar keratoplasty (DALK) in 1 patient, cyanoacrylate glue application in 1 patient, and awaiting DALK/PKP in three patients, 4 patients showing no signs of increased thinning.

**Conclusion:**

The incidence of corneal thinning after collagen cross-linking is less but fulminant, requiring timely and appropriate management to prevent visual complications.

## Introduction

Keratoconus (KC) is a bilateral, degenerative, non-inflammatory, and ectatic cornea disorder, characterized by corneal steepening, thinning, corneal scarring, and visual distortion [1]. Before collagen cross-linking (CXL), studies reported that 35% of cases eventually required corneal transplantation [2]. Spoerl and Seiler, from the University of Dresden, first postulated the treatment of ectatic corneal disorders is done with Riboflavin and ultraviolet A [3-6]. It creates free radicals that induce new chemical bonds causing collagen cross-linking, which causes mechanical stiffness. In 2003, Wollensak et al. [7] reported CXL as a potential treatment for halting the progression of keratectasia and alleviating the need for corneal transplantation in keratoconus. While clinical studies indicate that it is a safe procedure with few sight-threatening complications, adverse events can occur. Corneal thinning and corneal perforation are a rare complication but are sight-threatening and may require emergency keratoplasty. Few studies have been presented previously about post-CXL thinning. Therefore, we have tried to study a series of cases to understand the reason and course of corneal thinning and melting post-CXL treatment.

## Methods

The study was conducted at a tertiary eye care center in southern India from January 2011 to January 2017, adhering to the tenets of the Declaration of Helsinki. The case series was retrospective and observational. The institutional reviewer committee approved the study and the subjects gave informed consent. The study included patients who presented with corneal thinning and had CXL treatment before. Patients presenting with trauma-associated corneal perforation and patients with corneal thinning who did not undergo CXL treatment were excluded from the study. As the progression of keratoconus causes hydrops and not thinning, hydrops patients were also excluded from the study.

The demographic data, details about CXL treatment, preoperative corneal thickness, and postoperative vision were obtained from electronic medical records (EMR). After CXL treatment, the follow-ups were monthly for three months, at 3 months for one year, and yearly afterward. For the presenting complaints, a complete ophthalmic evaluation including a detailed history, slit lamp examination, fundus examination with indirect ophthalmoscopy, and assessment of best corrected visual acuity was done. Corneal thickness was evaluated by tomography and AS-OCT (anterior segment ocular coherence tomography). Patients with associated systemic illness underwent appropriate tests. Corneal melting was defined as progressive thinning with loss of stromal mass. Appropriate treatment such as bandage contact lens and penetrating keratoplasty (PKP) was administrated, PKP being the main treatment option for severe corneal thinning. All the patients were called weekly until corneal thickness was stabilized and monthly afterward, during follow-up visits, patients were monitored for any signs of progression or complications. Stability or improvement in vision and corneal thickness were assessed as indicators of treatment efficacy. Patients who showed signs of worsening corneal thinning or vision deterioration were closely monitored for potential PKP intervention.

## Results

The cases of post-CXL corneal thinning were those presenting with corneal thinning in individuals who had previously undergone CXL treatment for keratoconus. In our study, we observed twelve patients who had undergone CXL treatment between January 2011 and January 2017 and were showing symptoms of corneal thinning at that moment. 2,347 CXL procedures were performed in our hospital during that period, resulting in an incidence rate of 0.51%. All the patients had standard CXL following the Dresden protocol. The time interval between CXL treatment and corneal thinning ranged from 5 to 12 years. The age range of patients who underwent CXL ranged from 15 to 31 years. The age of initial presentation with corneal thinning ranged from 21 to 36 years. The mean age of presentation was 28.5 years. An interesting observation was that out of 12 patients, 8 were females who were either pregnant or lactating, except for 2. Among the females, 2 were pregnant, 2 were lactating, and 1 was undergoing IVF treatment. Additionally, 1 female had associated diabetes mellitus (DM) and hyperthyroidism. The habit of itching was observed in 2 patients. None of these patients had a history of autoimmune disease or other ocular surface diseases, such as dryness and meibomitis (**Table 1**).

**Table 1:** Associated conditions

Associated conditions	No. of patients
Pregnant women	*2*
Lactating women	*2*
IVF treatment	*1*
DM and Hyperthyroidism	*1*
Habit of itching	*2*
Meibomitis	0
Dryness	*0*
ernal keratoconjunctivitis	*0*
Autoimmune disease	0

Most of the pa tients presented with visual impairments as their primary concern, while four patients reported experiencing pain. Among those four patients, one exhibited severe pain and redness, while another reported both pain and foreign body sensation in their eye. On slit lamp, the examined patients had a variety of corneal conditions, including descemetocele, corneal perforation with shallow anterior chamber, corneal ulcer with conjunctivitis and hypopyon, and epithelial defect with crystalline deposit. Additionally, several patients were found to have corneal thinning and scarring. In one patient presenting with corneal ulcer and hypopyon, corneal scrapping was sent for culture and they came positive for Streptococcus pneumonia. Blood sugar and thyroid levels were checked and had high levels in patients with a history of those conditions, the rest of the patients showed normal levels.

It was observed that the best corrected visual acuity (BCVA) post-CXL treatment ranged from 6/6 to 6/18. The best corrected visual acuity ranged from 6/9 to CF 2 meters (counting figures) at the presentation of corneal thinning and post-treatment BCVA ranged from 6/9 to 6/60 (**Table 2**).

**Table 2:** BCVA (best corrected visual acuity) changes at different times

	Post CXL treatment	At the corneal thinning stage	Post-treatment
**Case 1**	6/9	6/36	6/12
**Case 2**	6/18	CF 1 Mtr	6/12
**Case 3**	6/9	6/36	6/18
**Case 4**	6/6	6/24	6/18
**Case 5**	6/6	CF 3 Mtrs	6/60
**Case 6**	6/9	6/18	6/18
**Case 7**	6/9	6/9	6/9
**Case 8**	6/6	6/9	6/9
**Case 9**	6/12	6/18	6/18
**Case 10**	6/12	6/60	6/12
**Case 11**	6/6	6/9	6/9
**Case 12**	6/9	6/18	6/18

The corneal thinnest corneal thickness (TCT) data collected before CXL ranged from 414 to 482 µm, and no cases with a corneal thickness below 400 µm were observed. With the present complaints, all the patients exhibited corneal thinning, with the thinnest corneal thickness (TCT) ranging from 212 to 350 µm. Furthermore, one case was identified with corneal perforation and a shallow anterior chamber. The K max value ranged from 44.3D to 55.7D at the time of corneal thinning, pre CXL, K max ranged from 43.6D to 54.2D. No correlation or link between vision impairment and the thickness of the cornea, and no connection to any accompanying medical conditions was observed (**Table 3**).

**Table 3:** Present associated conditions, clinical findings, BCVA (best corrected visual acuity), TCT (thinnest corneal thickness), and K max

Cases	Associated conditions	Clinical findings	BCVA	TCT (micrometers)	K max (in diopters)
**Case 1**	Lactating mother	Corneal Thinning, haze	6/36	221	44.3
**Case 2**	Pregnant	Thinning Conjunctivitis, Hypopyon	CF 1 Mtr	214	53
**Case 3**	Nil	Thinning, haze	6/36	212	54.8
**Case 4**	Pregnant	Corneal Thinning	6/24	267	55.7
**Case 5**	Lactating mother	Descemetocele	CF 3 Mtrs	241	48.7
**Case 6**	IVF treatment	Corneal Thinning	6/18	284	48
**Case 7**	DM, hyperthyroidism	Corneal Thinning	6/9	291	46.6
**Case 8**	Nil	Corneal Thinning	6/9	279	45
**Case 9**	Nil	Perforation, Shallow AC	6/18	0	-
**Case 10**	Eye rubbing	Epithelial Defect with Crystalline Deposit	6/60	351	45.3
**Case 11**	Nil	Corneal Thinning	6/9	215	47.8
**Case 12**	Eye rubbing	Corneal Thinning	6/18	290	46.8

**Fig. 1:**
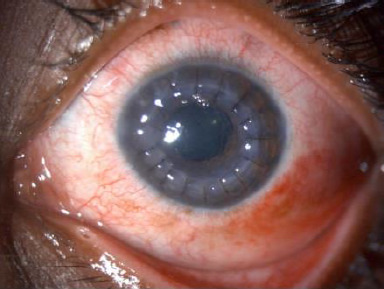
Slit lamp image of 1-week Post PKP surgery after corneal melting in a lactating mother

As an immediate treatment for supporting the cornea, BCL was placed in 2 patients, rose K2 contact lens was placed in 2 patients, and lubricant and antibiotic treatment was given. Two patients underwent PKP, while one underwent deep anterior lamellar keratoplasty (DALK). One patient necessitated prompt closure of their perforation and maintenance of the anterior chamber, therefore, cyanoacrylate glue closure was performed along with the administration of antibiotic treatment. In another patient, crystalline deposits were excised and the patient received steroid and antibiotic treatment. 5 individuals needed DALK. These individuals were monitored weekly and it was observed that the deterioration of their corneal structures stopped in two patients. One of these patients was a lactating mother who experienced a cessation of thinning after discontinuing lactation (**Fig. 1**).

Another patient, who was receiving IVF treatment, experienced the same outcome after putting an end to this treatment, as the IVF center was situated far from her hometown. 3 patients were awaiting DALK. Despite having a thinnest corneal thickness of 215, 1 patient was content with their visual acuity, which was 6/9. All patients were closely monitored and under regular follow-ups.

## Discussion

CXL is the first treatment modality to halt the progression of keratoconus, designed to slow, stabilize, or even possibly reverse the progression of corneal ectasia in the early phase of KC [2]. The treatment causes new collagen cross-linkages that strengthen the cornea. Many clinical trials have been conducted, which found that CXL is a very safe procedure with minimal complications, but is not devoid of complications. Post-CXL treatment, corneal thinning occurrence or melting is an infrequent complication. This study aimed to analyze a case set with this rare complication. The objective was to determine the demographic distribution, associated conditions, causes of corneal thinning, and condition treatment.

The study demonstrated that all patients underwent the standard Dresden protocol, and each patient exhibited a corneal thickness above 400 µm. The study revealed a gap of several years before the development of corneal thinning. Interestingly, female patients, especially those who were pregnant or lactating, were found to be at a higher risk for post-CXL corneal thinning. Eye-rubbing was also identified as a contributing factor in these cases. No other associations like dryness, meibomitis autoimmune conditions were observed. This study has demonstrated a multitude of distinct clinical presentations. No correlation has been observed between the patient’s visual acuity, corneal thinning, and their associated condition. Out of the twelve patients, most required either PKP or DALK procedures, while three opted for PKP/DALK, and two chose to use spectacles due to their visual comfort. Moreover, it is noteworthy that two of the patients experienced a significant halt in corneal thinning after the cessation of lactation and IVF treatment.

The causes for thinning post-CXL treatment in pregnant, lactation, and undergoing IVF treatment were studied. We assumed that the hormonal factors during these periods, which acted on the cornea were mainly responsible for thinning and increasing the risk of infection. It is a known fact that hormonal factors act on the cornea. Estrogen receptors were present on the cornea. In pregnancy and lactation, estrogen levels increase. It causes an increase in the level of matrix metalloproteinases (MMP), collagenases, and proteases, all of which have proteolytic activity on the cornea, leading to reduced biomechanical stability, corneal thinning, and perforation. Curry TE Jr et al. [8] noted an increase in metalloproteinases (MMPs), especially MMP-9, by trophoblastic cells during the first trimester of pregnancy, caused by the human chorionic gonadotropin hormone release. Studies state the same mechanism occurs even after CXL in pregnancy and lactation. Hafezi et al. [9] described a case of keratectasia diagnosis during pregnancy that recurred during a second pregnancy despite corneal CXL. Kodavoor SK et al. [10] described a case of corneal melt 7 years post CXL treatment during pregnancy and attributed such changes to hormonal changes during pregnancy and UV radiation. Similarly, in in-vitro fertilization (IVF) treatment, ovaries are stimulated for follicle development, which releases a high amount of estrogen, which has a thinning effect on the cornea. We had a patient who had a stable cornea after CXL for many years and presented with severe corneal thinning after 3 months of starting IVF and thinning stopped after stopping IVF treatment.

An associated finding of eye rubbing was seen in 2 patients. Balasubramanian et al. [11] observed that rubbing of the eyes resulted in elevated levels of inflammatory cytokines and MMP in tears of both healthy eyes and eyes with keratoconus. In our study, the patients were cotton industry workers, who were exposed to cotton fibers and had irritating eyes and a tendency to regularly rub their eyes, which might be the underlying cause of ocular thinning, despite having undergone CXL treatment.

**Fig. 2:**
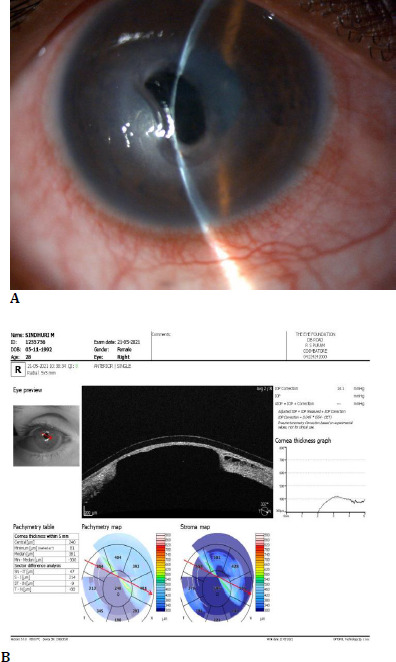
**A**. Severe corneal thinning after 6 years post CXL, in a 9-month pregnant woman; **B**. corresponding AS OCT showing the same. AS-OCT = anterior segment optical coherence tomography

In one of our patients, who was in the 9th month of her pregnancy, an occurrence of hormonal changes and infection existed concomitantly, resulting in a rapid deterioration of the corneal thinning (**Fig. 2 A, B**). This necessitated immediate PKP treatment, which was carried out after the delivery of the child.

One patient presented with full corneal perforation, no associated condition was present, as he had good vision, and he did not come to the hospital until the perforation happened. A patient presented 8 years post-CXL with a crystalline plaque over the corneal thinning. Following plaque removal and antibiotic-steroid treatment, the thinning has ceased. It is plausible to consider this a sterile infiltrate case combined with thinning. There was one similar study reported before, by Arora et al. [12], of a

10-year-old child with severe VKC, who developed a shield ulcer with sterile infiltrates post-conventional CXL and received intensive steroid therapy but showed no improvement. Slit-lamp examination highlighted a thick plaque covering the ulcer surface hindering the healing process. Debridement of the plaque and continued steroid therapy led to the resolution of the signs and symptoms in this patient. Koller et al. [13] reported sterile infiltrates in 8 eyes (7.6 %) of 117 cases resolving within 4 weeks with topical dexamethasone 0.1% treatment.

Another important finding was that all the patients in the study underwent a 30-minute standard Dresden protocol. Kandel et al. [14] observed in their study that while accelerated corneal collagen cross-linking (aCXL) resulted in greater thinning than the standard CXL protocol (sCXL), no statistically significant difference in the change of mean corneal thickness (MCT) existed between the two methods. Other studies have shown that sCXL causes greater thinning than aCXL postoperatively, but the differences were also not statistically significant in each case [15,16]. Another study, by Daizong Wen et al. [17], concluded that accelerated CXL possibly showed a better ability to keep CCT and protect corneal endothelial cells than standard CXL. As our study was small, we cannot conclusively attribute the incidence of corneal thinning to adherence to the standard CXL protocol.

Unregulated UV exposure and pre-CXL corneal thinning < 400 micrometers are risk factors for post-CXL corneal thinning. Thus, CXL is not recommended for patients whose corneas are thinner than 400 μm [6]. Because 85% to 90% of the UVA radiation is absorbed in the anterior 400 μm of the cornea, the procedure should not harm the patient’s corneal endothelium, lens, and retina [7]. In our study, no patients with a corneal thickness of less than 400 µm before undergoing CXL treatment existed. Lim et al. [18] also conducted a study that described UVA exposure as the cause of keratocyte death leading to a sub-lethal effect in the deeper stroma and causing thinning. Prabhakar et al. [19] reported stromal degeneration in three patients, highlighting the possible late effects of UVA irradiation as a cause for thinning.

Koller T et al. [13] stated that preoperative K max of more than 58D is also a risk of failure of CXL treatment. In our study, preoperative K max ranged between 44.3 D and 55.7 D. An age older than 35 when the CXL treatment was started was identified as a significant risk factor for complications [13]. In our study, the age range of our participants was found between 15-31 years. While we had one patient with an age of 31, it would not be appropriate to attribute this increased age as the only cause for thinning. We can attribute corneal thinning in one of our participants to the risk factor of diabetes mellitus and hyperthyroidism, as corneas of diabetic patients are susceptible to nerve damage, hypoesthesia, and subsequent neurotrophic cornea development, delayed corneal wound healing, delayed reepithelization, persistent epithelial defect and increased corneal expression of MMP [20-22].

## Conclusions

Based on the discussion in our study, hormonal changes, and eye rubbing were the primary contributing factors to corneal thinning, with diabetes mellitus as a risk factor in one patient and age of 31 as a risk factor in another. However, in 3 cases, the exact cause of corneal thinning remained unidentifiable.

In our study, the thinnest corneal thickness ranged from 212 µm to 351 µm, except for one patient who experienced full corneal perforation. It is important to note, that, despite severe corneal thinning, patient vision remained in a relatively good range (6/60 to 6/9), except for 2 cases. Although PKP/DALK was recommended for many patients, only three underwent the procedure. Most patients preferred to wear glasses.

The present study was limited due to a relatively small sample size. Additionally, after penetrating keratoplasty (PKP), the removed corneal button could not undergo histopathological examination due to logistical constraints. Despite the limitations, the study explored various presentation and treatment alternatives to address complications. It emphasized the significance of follow-ups, and timely and well-informed actions to overcome the complications. CXL is a safe procedure, however, it is important to acknowledge that even one instance of complications can lead to severe consequences, requiring significant interventions. Therefore, it is crucial to comprehend the underlying causes for such complications, to minimize the risks associated with the procedure. These findings highlight the importance of regular follow-ups after CXL treatment. In addition, it is crucial to educate female patients to schedule follow-up appointments during pregnancy and lactation periods. Moreover, the patient must be educated to avoid eye rubbing.
